# Mind–Body Interventions for Prediabetes Management: Traditional Chinese Exercise and Its Dual Effects on Metabolic Control and Psychological Well-Being—A Systematic Review and Meta-Analysis

**DOI:** 10.1155/jdr/8249301

**Published:** 2025-11-10

**Authors:** Yaoming Yang, Ran Chen, Xinbao Wang, Yuqian Yan, Tao Huang, Chunfang Wang, Guoyong Yu

**Affiliations:** ^1^Department of Preventive Treatment of Disease, Dongzhimen Hospital, Beijing University of Chinese Medicine, Beijing, China; ^2^School of Traditional Chinese Medicine, Beijing University of Chinese Medicine, Beijing, China; ^3^School of Acupuncture and Tuina, Chengdu University of Traditional Chinese Medicine, Chengdu, China

**Keywords:** blood lipid indicators, blood sugar indicators, prediabetes, psychological status, traditional Chinese exercise

## Abstract

**Aims:**

This study was aimed at evaluating the effectiveness of traditional Chinese exercises (TCEs) in improving both physical and psychological health in individuals with prediabetes.

**Methods:**

Randomized controlled trials were systematically searched in the Cochrane Library, PubMed, Web of Science, Embase, China National Knowledge Infrastructure (CNKI), Wanfang, VIP, and the Chinese Biomedical Literature Database (CBM) up to August 28, 2025. Data from 22 trials involving 1854 participants were analyzed using Stata 15.0. The risk of bias was assessed with the Cochrane RoB 2.0 tool.

**Results:**

TCEs significantly improved fasting blood glucose (FBG), insulin resistance, glycated hemoglobin (HbA1c), 2-h postprandial blood glucose (2hPG), and several psychological outcomes compared with controls. Subgroup analyses showed consistent improvements in FBG at 3, 6, and 12 months, although the effect size and heterogeneity varied across time points. HbA1c and 2hPG improved at 3 and 6 months, while fasting insulin changes were significant only at 3 months. Baduanjin and Qigong were most effective for metabolic outcomes, whereas evidence for Taiji was limited and inconclusive, though some studies suggested potential benefits for HbA1c. Psychological benefits were observed across all exercise types.

**Conclusion:**

TCEs may enhance metabolic regulation and psychological well-being in individuals with prediabetes, potentially reducing the risk of progression to Type 2 diabetes. However, due to high heterogeneity and small sample sizes for some outcomes, these findings should be interpreted with caution. Large, well-designed multicenter trials are warranted to confirm these effects.

## 1. Introduction

Prediabetes mellitus (PreDM) is an intermediate stage between normal glucose regulation and diabetes, characterized by impaired glucose metabolism and elevated blood glucose levels. It is primarily associated with reduced insulin sensitivity (insulin resistance) and abnormal *β*-cell function. Individuals with PreDM have a substantially increased risk of developing diabetes, with approximately 5%–10% progressing to diabetes annually [1].

In China, an estimated 148.2 million people have PreDM, with a prevalence as high as 15.5% [2]. Without intervention, approximately 7% of individuals with impaired glucose tolerance (IGT) develop diabetes each year [3]. Globally, 589 million people are living with diabetes mellitus (DM), with one-quarter residing in China, giving the country the highest prevalence worldwide [4]. DM is a common endocrine disorder caused by chronic hyperglycemia resulting from insulin resistance or insufficient insulin secretion. Persistent hyperglycemia can damage multiple organs and tissues, leading to life-threatening complications and a significant decline in quality of life.

Currently, interventions for prediabetes primarily include lifestyle modifications and pharmacological treatments. Clinical trials have demonstrated that antidiabetic drugs such as metformin, thiazolidinediones, *α*-glucosidase inhibitors, and GLP-1 receptor agonists can reduce the risk of progression to diabetes in individuals with prediabetes [5]. However, concerns about cost, side effects, and adverse reactions often limit patient acceptance of pharmacological therapy, leading to low adherence and restricted implementation in clinical practice. Consequently, lifestyle interventions, such as dietary control and increased physical activity, remain the cornerstone of prediabetes management. Several studies have shown that lifestyle interventions significantly reduce risk factors such as fasting blood glucose (FBG) and body weight, thereby delaying the progression to diabetes and lowering its incidence [6, 7]. This represents a cost-effective, nonpharmacological strategy for diabetes prevention.

Aerobic exercise has been shown to enhance skeletal muscle glucose uptake and utilization, thereby improving insulin resistance and promoting glucolipid metabolism [8]. However, adherence to regular high-intensity exercise is challenging for many individuals. Low-intensity exercise is generally more acceptable and sustainable compared with high-intensity regimens [9, 10]. In addition, evidence from older adults indicates that combining structured exercise with nutritional supplementation can enhance functional outcomes and metabolic health, highlighting the potential benefits of multicomponent lifestyle interventions [11]. Traditional Chinese exercises (TCEs)—including Taiji, Baduanjin, and Qigong—are primarily low-intensity aerobic activities based on traditional Chinese medicine theories. They have gained global popularity due to their simplicity, accessibility, and dual benefits for physical and psychological well-being [12]. Qigong is a broad term encompassing mind–body practices that integrate breath control, physical postures, and mental focus. Taiji, although often categorized as a martial art, is frequently considered a dynamic form of Qigong due to its slow, flowing movements coordinated with breathing and intention. Baduanjin, literally translated as “Eight Pieces of Brocade,” consists of a standardized set of standing exercises that share Qigong's fundamental principle of cultivating vital energy (Qi). Some scholars regard Taiji and Baduanjin as distinct branches within the Qigong system, whereas others view them as independent traditions with unique historical and theoretical foundations. Distinguishing these practices from general Qigong allows for a more precise assessment of their specific effects on glycemic control and helps avoid potential confounding when diverse Qigong forms are aggregated in meta-analyses.

TCEs are simple and practical, making them highly feasible as interventions for individuals with prediabetes by improving adherence and compliance. A growing number of studies have reported that these exercises can improve blood glucose levels and insulin resistance. However, most existing reviews either focus on a single exercise type or fail to comprehensively summarize psychological outcomes [13, 14]. Therefore, there remains a need for a systematic review that integrates both metabolic and psychological indicators to provide a comprehensive evaluation of TCEs for prediabetes management.

To address this gap, we conducted a meta-analysis of randomized controlled trials (RCTs) investigating the effects of TCEs on individuals with prediabetes. The aim was to comprehensively evaluate the impact of these exercises on physical health, psychological well-being, and metabolic outcomes, thereby providing evidence-based support for the clinical application of TCEs in prediabetes management.

## 2. Methods

**Figure 1:**
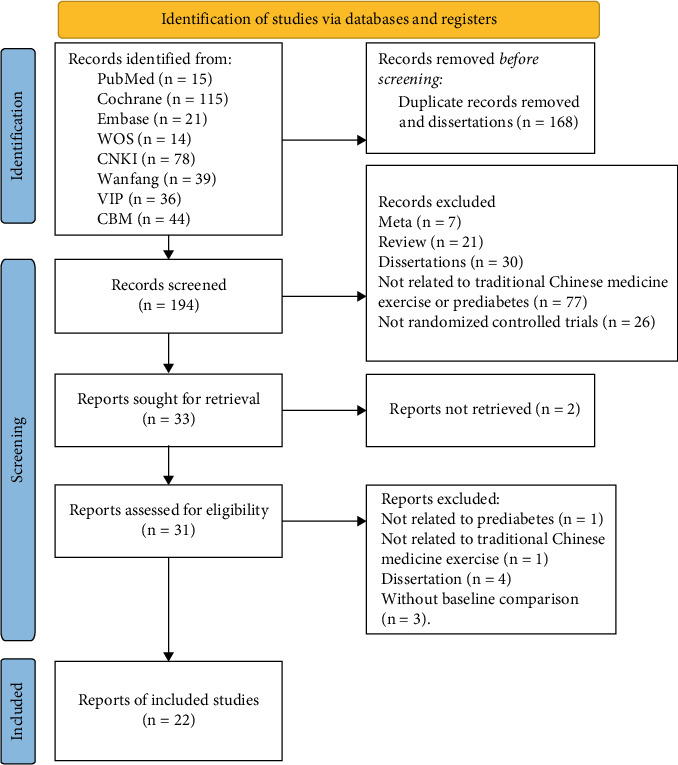
The process and results of literature screening.

This meta-analysis was conducted following the Cochrane Handbook for Systematic Reviews of Interventions (http://training.cochrane.org/handbook) and the Preferred Reporting Items for Systematic Reviews and Meta-Analyses (PRISMA) guidelines [15]. The study protocol was registered in PROSPERO (ID: CRD42023442189). A detailed flow diagram of the study selection process is shown in Figure 1.

### 2.1. Inclusion and Exclusion Criteria

#### 2.1.1. Participants

The participants of this study include the following:

1. Adults aged ≥ 18 years.
2. Met the WHO 1999 criteria for prediabetes diagnosis [16], which defines prediabetes as impaired fasting glucose (IFG), IGT, or both (IFG+IGT).

#### 2.1.2. Intervention Measures

The experimental group received interventions involving TCEs, including Qigong, Baduanjin, and Taiji. Specific Qigong forms included rejuvenating exercise, Shaolin Internal Qigong, Yijin Jing, and Wuqin Xi. The control group used different approaches, such as maintaining their original lifestyle, walking, or participating in a comprehensive community-based intervention. Community-based programs generally consisted of health education (e.g., lifestyle counseling, exercise guidance, and diabetes self-management education) and dietary management (e.g., individualized dietary plans or standardized nutritional guidelines).

The content, intensity, and frequency of these interventions varied across studies, which may have contributed to heterogeneity. To address this, sensitivity and subgroup analyses were performed when data permitted, and the potential effects of these variations were considered during the interpretation of the results. The intervention duration ranged from 1 to 12 months.

#### 2.1.3. Outcome Indicators

The outcome indicators are as follows:

*Primary outcomes*: FBG, oral glucose tolerance test (OGTT), insulin resistance index, glycated hemoglobin (HbA1c), and 2-h postprandial glucose (2hPG).

*Secondary outcomes*: incidence of progression to Type 2 diabetes mellitus (T2DM), fasting insulin (FINS), mental health indicators, body mass index (BMI), waist-to-hip ratio (WHR), and blood lipid levels.

#### 2.1.4. Research Type

Only RCTs were included.

#### 2.1.5. Exclusion Criteria

Exclusion criteria include participants who did not have prediabetes or other diseases of interest; studies with unavailable or incomplete data; non-RCTs, reviews, pathological studies, animal experiments, academic dissertations, or conference papers; and literature not published in Chinese or English.

### 2.2. Retrieval Strategy

Two researchers independently performed a comprehensive literature search across eight electronic databases from their inception to August 28, 2025: the Cochrane Library, PubMed, Web of Science, Embase, Wanfang, VIP, China National Knowledge Infrastructure (CNKI), and the Chinese Biomedical Literature Database (CBM). Both controlled vocabulary terms (e.g., MeSH) and free-text keywords were used to ensure a thorough search. Detailed search strategies for each database are provided in Appendix S1.

### 2.3. Literature Screening and Data Extraction

Two researchers independently screened studies according to the predefined inclusion and exclusion criteria. Duplicate records were removed using EndNote X9. Titles and abstracts were then screened, followed by full-text reviews to identify eligible studies.

Data were independently extracted by both researchers and cross-checked for accuracy. Any discrepancies were resolved through discussion, and when consensus could not be reached, a third researcher was consulted. Extracted information included the study title, first author, publication year, country, study type, sample size, sex distribution, intervention details, study duration, and outcome indicators.

### 2.4. Bias Risk Assessment in Included Studies

The methodological quality of the included studies was independently evaluated by two reviewers using the Cochrane Risk of Bias 2.0 (RoB 2.0) tool. This tool examines five domains: the randomization process, deviations from intended interventions, missing outcome data, outcome measurement, and the selection of reported results. Studies rated as high risk in any domain were considered to have an overall high risk of bias. Any discrepancies between reviewers were resolved through discussion, and, when consensus could not be reached, a third reviewer adjudicated the decision. Predefined decision rules were established prior to assessment, including a preference for information reported in trial protocols or registrations whenever available. Based on these evaluations, studies were classified as having low risk, some concerns, or high risk of bias [17].

### 2.5. Statistical Method

This study analyzed multiple outcome indicators, including FBG, insulin resistance, HbA1c, 2hPG, depression scores, high-density lipoprotein cholesterol (HDL-C), low-density lipoprotein cholesterol (LDL-C), total cholesterol (TC), triglycerides (TGs), BMI, WHR, and FINs. Data were analyzed using Stata Version 15.0. Continuous variables were pooled using the standardized mean difference (SMD) with 95% confidence intervals (CIs), whereas dichotomous variables were combined using relative risk (RR) with 95% CI.

Heterogeneity was assessed using the *Q* statistic and *I*^2^ test, with *I*^2^ values greater than 50% indicating substantial heterogeneity. A fixed-effects model was applied when *p* > 0.1 and *I*^2^ ≤ 50%; otherwise, a random-effects model was employed. To explore potential sources of heterogeneity, subgroup analyses were conducted based on intervention duration (3, 6, and 12 months), intervention type (Qigong, Baduanjin, or Taiji), and type of control group (maintaining their original lifestyle, comprehensive community-based intervention, or walking). Sensitivity analyses were also performed to assess the robustness of the results. Publication bias was initially evaluated using funnel plots, and in cases of observed asymmetry, further statistical testing was conducted using the “metabias” command in Stata.

### 2.6. Data Handling for Multiarm Trials

For studies with multiple intervention arms (e.g., Baduanjin, walking, and no-intervention control), data were managed to prevent double-counting participants in the meta-analysis. When two or more experimental arms involved similar interventions, their outcomes were combined using the standard formulas recommended in the Cochrane Handbook. In cases where multiple control groups were reported, sample sizes were either split proportionally or only the most appropriate comparator was included, depending on the context. These procedures ensured that each participant contributed data to the meta-analysis only once, thereby minimizing bias and preserving the integrity of the pooled estimates.

### 2.7. Data Extraction

Two reviewers independently extracted data from the included studies using a standardized form. For continuous outcomes, mean values and standard deviations (SDs) were collected. When only standard errors (SEs) or 95% CIs were reported, these were converted to SDs using standard formulas. For studies reporting medians with interquartile ranges (IQR) or ranges, means and SDs were estimated using established statistical methods. Any discrepancies between reviewers were resolved through discussion, and a third reviewer adjudicated disagreements that remained unresolved. Extracted data included sample sizes, participant characteristics, intervention details, and outcome measures.

## 3. Results

### 3.1. Literature Search Results

A total of 362 articles were retrieved from various databases and imported into EndNote X9. After removing 168 duplicates and screening titles and abstracts, irrelevant studies were excluded. Full-text review of the remaining articles led to the exclusion of 172 studies that did not meet the inclusion criteria. Ultimately, 22 articles were included in the analysis [18–39] (Figure 1).

### 3.2. Basic Characteristics of the Included Literature

This meta-analysis included 22 RCTs comprising 1854 participants, of whom 771 were assigned to the experimental group and 1055 to the control group [18–39]. Among these studies, eight investigated Qigong, 11 examined Baduanjin, and three focused on Taiji. Control interventions consisted of comprehensive community-based intervention (11 studies), walking exercises (12 studies), and maintaining their original lifestyle (7 studies). The detailed characteristics of the included studies are provided in Table S1.

### 3.3. Quality Assessment

The risk of bias in the 22 included studies was assessed using RoB 2.0, and both a risk-of-bias ratio plot (Figure S1) and a methodological quality summary (Figure S2) were generated. All included studies were RCTs. Among them, 10 studies demonstrated higher methodological quality [20–22, 27, 29, 31, 32, 35, 37, 39]. Random sequence generation and blinding were generally assessed as low risk. Allocation concealment, completeness of outcome data, selective reporting, and other potential biases were also largely rated as low risk. Additionally, one study was rated as having some concerns (neutral risk) due to issues in measurement of the outcome and selection of the reported result [33]. However, 11 studies did not report allocation concealment [18, 19, 23–26, 28, 30, 34, 36, 38]. Six studies reported participant dropout, but the dropout data were not included in the final analysis [20, 27, 29, 32, 33, 36]. Other studies did not report losses to follow-up. Reporting of study results was complete in all studies. None of the studies reported adverse events, indicating an overall high quality of the included studies.

### 3.4. Meta-Analysis Results

Subgroup analyses were conducted to explore the heterogeneous effects of TCEs interventions in prediabetes management. First, the impact of prolonged exposure (longest duration subgroup) on metabolic outcomes (FBG, HbA1c, FINS, insulin resistance, and 2hPG), lipid profiles (BMI, WHR, HDL-C, LDL-C, TC, and TG), and psychological outcomes (depression, anxiety, somatization, interpersonal sensitivity, paranoia, and psychotic factor scores) was examined. Second, a duration-stratified subgroup analysis was performed to investigate potential dose–response relationships between intervention length and efficacy. Third, intervention modalities were compared between experimental and control groups to elucidate potential mechanisms underlying differences in efficacy and to assess the relative benefits of TCEs over alternative interventions. Collectively, these analyses were aimed at identifying optimal exercise protocols by integrating intervention intensity (duration) and implementation strategies (modality) for comprehensive prediabetes management.

### 3.5. Meta-Analysis Results of FBG

**Figure 2:**
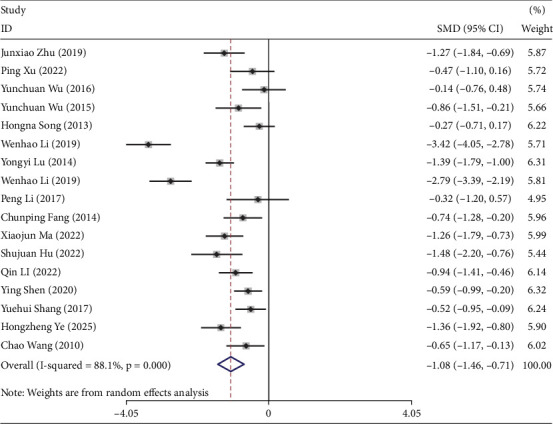
The forest plot under the maximum intervention duration of FBG.

Seventeen studies reported the effects of TCEs interventions on FBG levels in prediabetic participants [19–21, 23–30, 32–36, 38], including 635 participants in the experimental groups and 726 in the control groups. The overall analysis indicated that TCEs interventions significantly improved FBG levels in prediabetic patients (*I*^2^ = 88.1%, SMD = −1.08, 95% CI [−1.46, −0.71], *p* < 0.05) (Figure 2).

We further analyzed outcomes according to intervention duration. Studies with 1-, 2-, and 4-month durations were excluded from this analysis. For the 3-month (*I*^2^ = 76.9%) and 6-month (*I*^2^ = 91.6%) subgroups, a random-effects model was applied, whereas a fixed-effects model was used for the 12-month subgroup (*I*^2^ = 21.3%). TCEs improved FBG levels compared with controls across all durations (Table 1). Notably, heterogeneity was substantially lower in the 12-month subgroup than in the 3- and 6-month subgroups. This may be attributed to several factors: fewer studies contributed to the 12-month analysis, which can reduce apparent heterogeneity; longer interventions may have been implemented more consistently across studies, yielding more uniform effects; and only higher quality studies with standardized protocols may have extended to 12 months, reducing variability between trials. These findings suggest that intervention duration not only affects efficacy but may also influence the consistency of observed outcomes, which should be considered when interpreting the results.

Subgroup analysis by intervention type in the experimental groups indicated that TCEs significantly improved FBG (SMD = −1.08, 95% CI [−1.46, −0.71], *p* < 0.05), whereas the Taiji subgroup showed no significant difference (SMD = −1.51, 95% CI [−3.08, 0.07], *p* > 0.05) (Table 1).

Further subgroup analysis by control group demonstrated that TCEs outperformed groups maintaining a regular lifestyle, community-based interventions, and walking (SMD = −0.98, 95% CI [−1.30, −0.66], *p* < 0.05) (Table 1).

### 3.6. Meta-Analysis Results of Insulin Resistance Index

**Figure 3:**
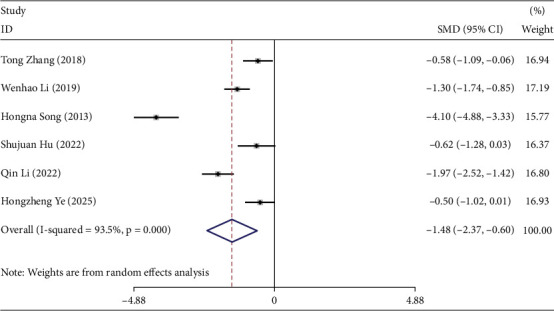
Forest plot under the maximum intervention duration of insulin resistance index.

Six studies reported the effects of TCEs interventions on the insulin resistance index in prediabetic participants [18, 21, 26, 27, 33, 36], including 207 participants in the experimental groups and 290 in the control groups. Overall, TCEs significantly improved insulin resistance compared with control interventions (*I*^2^ = 93.5%, SMD = −1.48, 95% CI [−2.37, −0.60], *p* < 0.05) (Figure 3).

A subgroup analysis based on intervention duration was conducted, excluding two studies with 1- and 9-month durations. Using a random-effects model, no significant difference in insulin resistance was observed between experimental and control groups (Table S2).

Subgroup analysis by intervention type (Table S2) indicated that TCEs significantly improved insulin resistance (SMD = −1.48, 95% CI [−2.37, −0.60], *p* < 0.05), whereas no significant effect was found in the Taiji subgroup. Analysis by control group (Table S2) showed that TCEs outperformed controls overall (SMD = −1.35, 95% CI [−2.13, −0.57], *p* < 0.05), while no significant differences were observed in comparisons with community-based interventions or walking.

### 3.7. Meta-Analysis Results of HbA1c

**Figure 4:**
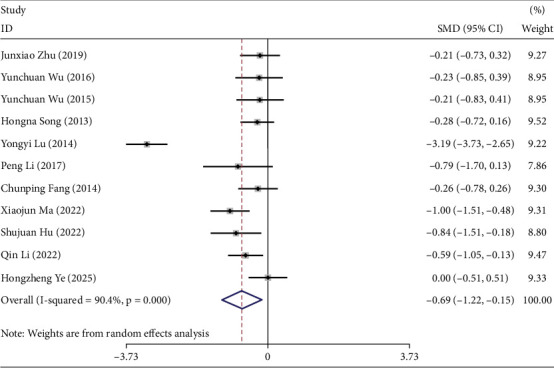
Forest plot under the maximum duration of intervention for glycated hemoglobin.

Eleven studies reported the effects of TCEs interventions on HbA1c in prediabetic participants [19–21, 24–26, 28, 30, 32, 33, 36], including 398 participants in the experimental groups and 446 in the control groups. Overall, TCEs significantly improved HbA1c levels compared with controls (*I*^2^ = 90.4%, SMD = −0.69, 95% CI [−1.34, −0.15], *p* < 0.05) (Figure 4).

A combination of fixed- and random-effects models was used according to intervention duration. For 3- and 6-month interventions (*I*^2^ = 0.0%), a fixed-effects model was applied, whereas a random-effects model was used for the 12-month intervention (*I*^2^ = 96.2%). The results showed that participants practicing TCEs exhibited improvements in HbA1c at 3 and 6 months compared with controls, but no statistically significant difference was observed at 12 months (Table S3).

Subgroup analysis by intervention type in the experimental groups (Table S3) indicated that TCEs significantly improved HbA1c compared with controls (SMD = −0.69, 95% CI [−1.22, −0.15], *p* < 0.05). No significant differences were observed between the Taiji and Qigong subgroups and the control group.

Further subgroup analysis by control group (Table S3) demonstrated that TCEs outperformed controls in improving HbA1c (SMD = −0.60, 95% CI [−1.02, −0.18], *p* < 0.05), whereas no significant differences were found when compared with community-based interventions or walking.

### 3.8. Meta-Analysis Results of 2hPG

**Figure 5:**
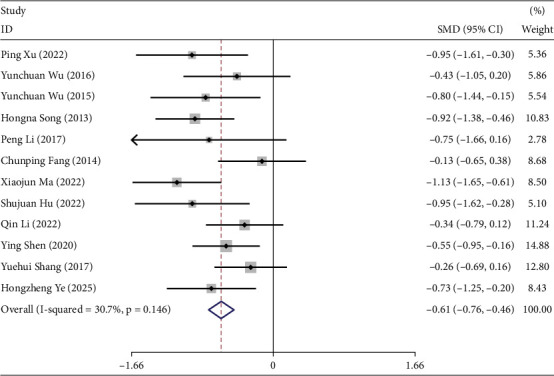
Forest plot under the maximum intervention duration of 2hPG.

Twelve studies investigated the effects of TCEs interventions on 2hPG levels in prediabetic participants [20, 21, 23–26, 30, 32–36], including 425 participants in the experimental groups and 472 in the control groups. Overall, TCEs significantly improved 2hPG levels compared with controls (*I*^2^ = 30.7%, SMD = −0.61, 95% CI [−0.76, −0.46], *p* < 0.05) (Figure 5).

Subgroup analyses were conducted based on intervention duration. For 3- and 6-month interventions (*I*^2^ = 0.0%), a fixed-effects model was applied, whereas a random-effects model was used for the 12-month intervention (*I*^2^ = 86.0%). TCEs improved 2hPG at 3 and 6 months, but no significant difference was observed at 12 months (Table S4).

Subgroup analysis by intervention type (Table S4) indicated that TCEs significantly improved 2hPG compared with controls (SMD = −0.61, 95% CI [−0.76, −0.46], *p* < 0.05).

Further analysis by the control group (Table S4) demonstrated that TCEs were more effective than controls in improving 2hPG (SMD = −0.60, 95% CI [−0.83, −0.37], *p* < 0.05).

### 3.9. Meta-Analysis Results of OGTT

**Figure 6:**
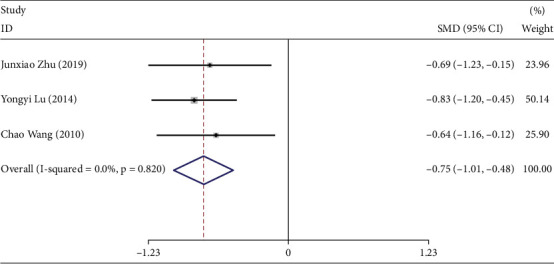
Forest plot under the maximum intervention duration of OGTT.

Three studies reported the effects of TCEs interventions on OGTT in prediabetic participants [19, 28, 38], including 118 participants in the experimental groups and 118 in the control groups. The results of this analysis are presented in Figure 6.

The pooled effect size was calculated using a fixed-effects model (*I*^2^ = 0.0%, *p* = 0.681). The analysis indicated that TCEs significantly improved OGTT compared with controls (SMD = −0.75, 95% CI [−1.01, −0.48], *p* < 0.05).

As the studies included in this analysis implemented the same intervention across both control and experimental groups, no further subgroup analysis was conducted.

### 3.10. Meta-Analysis Results of Secondary Outcome Indicators

In the analysis of secondary outcomes based on intervention duration (Table S5), TCEs demonstrated greater improvements than controls in depression, anxiety, somatization, TC, TG, WHR, and the incidence of conversion to T2DM. The corresponding results are presented in the supporting information.

Subgroup analysis revealed duration-specific effects. At 3 months, the experimental group showed improvements in TC and FINS; at 6 months, significant improvements were observed in LDL-C, TC, and WHR; and at 12 months, the experimental group outperformed controls in depression, anxiety, and TC (Table S6). Further subgroup analysis by intervention type in the experimental groups (Table S7) indicated that TCEs, particularly Baduanjin, significantly improved depression, anxiety, TG, TC, and WHR. Qigong and Taiji also demonstrated benefits for physical health, although insufficient data limited more detailed analysis.

Analysis by the control group (Table S8) showed that TCEs outperformed community-based interventions in improving depression, anxiety, somatization, TC, and WHR. Additionally, the experimental group exhibited greater improvements in TG and TC compared with participants maintaining their usual lifestyle and in anxiety, BMI, and WHR compared with the walking group.

### 3.11. Sensitivity Analysis and Publication Bias

For FBG, sensitivity analysis was conducted by sequentially excluding each study. No significant changes were observed at 3, 6, or 12 months, confirming the robustness of the meta-analysis results. Egger's test indicated no evidence of publication bias (*p* > 0.05), and none of the included studies showed bias across the assessed indicators (Table S9).

## 4. Discussion

Prediabetes represents a critical stage in the progression to Type 2 diabetes, and timely interventions are essential to prevent its transition. TCEs, including Baduanjin, Qigong, and Taiji, offer an integrative approach that combines aerobic activity, meditation, and mind–body regulation. Our meta-analysis of 22 RCTs involving 1854 prediabetic participants demonstrates that these interventions improve both metabolic and psychological outcomes, with effects varying according to exercise modality and specific outcome measures.

Baduanjin consistently improved glycemic parameters, including FBG, 2hPG, insulin resistance, and HbA1c, likely due to its moderate intensity, rhythmic movements that enhance skeletal muscle glucose uptake and improve insulin sensitivity [40–42]. Qigong also contributed to glycemic control, although to a slightly lesser extent, and provided additional psychological benefits through meditative focus and breath regulation [43–45]. Taiji, while less effective for short-term metabolic regulation, may offer complementary benefits for long-term cognitive function, balance, and mental health, reflecting its emphasis on mindful, complex movements. These findings suggest that exercise prescriptions for prediabetic patients should be tailored to specific health goals, with Baduanjin and Qigong targeting short- to medium-term glycemic control and Taiji supporting long-term mental and physical well-being.

In addition to glucose metabolism, TCEs also influenced lipid profiles and body composition. Baduanjin showed the most consistent improvements in TC, TG, and WHR, likely due to its aerobic characteristics and ability to increase energy expenditure [46, 47]. Qigong conferred modest benefits, whereas Taiji had limited effects on lipid metabolism and anthropometric measures, potentially reflecting its lower cardiovascular intensity. These findings underscore the importance of tailoring exercise interventions to patient characteristics and metabolic goals. For individuals with obesity or dyslipidemia, aerobic-oriented exercises such as Baduanjin may be preferable to optimize lipid and weight control outcomes.

Our findings suggest that TCEs may exert a dual effect, simultaneously improving physiological and psychological outcomes in individuals with prediabetes. Physiologically, slow and rhythmic movements combined with controlled breathing, as seen in Baduanjin and Qigong, may help regulate the autonomic nervous system (ANS) by enhancing parasympathetic activity and reducing sympathetic overdrive. This autonomic balance, together with potential downregulation of the hypothalamic–pituitary–adrenal (HPA) axis, can improve insulin sensitivity, glucose metabolism, and lipid regulation [48, 49]. Psychological improvements are also clinically relevant. Individuals with prediabetes in China may experience anxiety symptoms [50, 51], while TCEs have been shown to enhance psychological well-being in this population. This meta-analysis confirms that such exercises can effectively reduce depression, anxiety, and somatization [52, 53]. These benefits may arise not only from neurophysiological effects (ANS and HPA axis regulation) but also from behavioral mechanisms, including increased self-efficacy and social support derived from group practice [54]. Psychological outcomes also varied by exercise type. Baduanjin was particularly effective in reducing depression and anxiety, potentially due to its structured breathing patterns and accessible, easy-to-learn movements that promote rapid stress relief. Qigong's meditative emphasis further enhances calming effects on the nervous system, benefiting individuals experiencing high psychological stress. Although Taiji did not show strong short-term psychological improvements, its mindful, prolonged practice may contribute to gradual enhancements in cognitive and emotional regulation. These differential effects underscore the importance of tailoring exercise prescriptions to the psychological needs of prediabetic patients. Overall, our results suggest that TCEs provide an integrated approach to prediabetes management, addressing both metabolic dysregulation and psychological distress through complementary physiological and behavioral pathways.

Compared with previous reviews [13, 14], this meta-analysis included additional RCTs published after the time frame of those studies, increasing the total sample size to 1854 prediabetic participants. We also evaluated a broader range of outcome indicators, encompassing not only FBG, glycosylated hemoglobin, and insulin resistance but also psychological outcomes (depression, anxiety, and somatization) and anthropometric measures (BMI, WHR, and weight), which were underrepresented in prior reviews. These enhancements enabled subgroup analyses by intervention type, duration, and control group, providing more nuanced insights into the differential effects of Baduanjin, Qigong, and Taiji on both metabolic and psychological parameters. Consequently, our findings suggest that Baduanjin and Qigong are particularly effective for short- to medium-term glycemic control and psychological improvement, whereas Taiji may contribute more to long-term overall health, offering a more comprehensive understanding than previous reviews.

Heterogeneity across studies was influenced by intervention duration, exercise modality, participant characteristics (e.g., age, baseline glucose levels, and comorbidities), and study quality. Notably, 12-month interventions exhibited lower heterogeneity, which may reflect fewer included studies, more consistent intervention protocols, or higher quality trials. Subgroup analyses by intervention type, duration, and control group partially reduced heterogeneity but did not fully account for it. These findings suggest that future trials would benefit from standardized intervention protocols for specific TCEs (e.g., Baduanjin), clearly defined active control groups (e.g., health education or structured walking), and head-to-head comparisons across different exercise modalities. Ideally, such studies should include adequate sample sizes, multicenter recruitment, and long-term follow-up to clarify relative efficacy, reduce heterogeneity, and provide more precise guidance for clinical practice, building on the evidence established by current research. Sensitivity analyses indicated that the overall findings were robust, suggesting that TCEs may reduce fasting glucose and FINS, improve insulin resistance, and alleviate anxiety.

Overall, TCEs represent a safe, low-cost, and culturally adaptable strategy for prediabetes management, simultaneously improving metabolic and psychological outcomes. Clinically, these interventions may serve as first-line nonpharmacological strategies to delay progression to diabetes, particularly in populations with limited access to medical resources or at risk of poor medication adherence. Policymakers and healthcare providers should consider integrating TCEs programs into community-based diabetes prevention initiatives. Despite these promising findings, several limitations should be noted. First, the broad variety of TCEs makes it difficult to isolate the effects of specific modalities beyond Baduanjin and Qigong. Second, the absence of sham controls limited blinding. Third, some subgroup analyses included few studies, which may affect reliability. Most included studies were conducted in Asian populations, highlighting the need for more diverse participant inclusion. Additionally, variations in intervention duration, intensity, and frequency could not be fully standardized, potentially influencing outcomes. Longer term interventions such as Wuqinxi and Taiji, which require gradual skill acquisition and sustained practice, may yield different benefits compared with shorter duration programs like Baduanjin or Qigong. Future RCTs should stratify participants by intervention length and systematically evaluate dose–response relationships to establish optimal training durations and refine clinical guidance.

In conclusion, Baduanjin and Qigong demonstrate short- to medium-term improvements in glycemic control and psychological outcomes, whereas Taiji may contribute to long-term mental and physical health benefits. Tailored exercise prescriptions that consider patient-specific health priorities, intervention modality, and duration could maximize therapeutic potential, supporting the integration of TCEs into comprehensive prediabetes management strategies.

## 5. Conclusion

TCEs, particularly Baduanjin and Qigong, may improve glycemic control, insulin sensitivity, and psychological well-being in individuals with prediabetes, whereas Taiji appears to provide more gradual, long-term benefits. However, the overall evidence is limited by high heterogeneity, variable intervention protocols, small sample sizes, and the predominance of studies conducted in Asian populations. Future high-quality, multicenter RCTs with standardized exercise protocols, clearly defined control groups, and longer follow-up periods are needed to confirm these findings and clarify the relative efficacy of different exercise modalities. Despite these limitations, TCEs represent a promising, culturally adaptable, and low-cost strategy for the prevention and management of prediabetes.

## Figures and Tables

**Table 1:** The traditional Chinese medicine exercise's impact on fasting blood glucose (FBG) in patients with prediabetes across different subgroups.

**Subgroup factors**	**K** ** (the number of studies)**	**SMD (the pooled effect size)**	**95% CI**	**I** ^2^ ** value**	**p** ** value**
3 months	8	−0.72	(−1.16, −0.29)	76.9%	< 0.05
6 months	8	−1.11	(−1.75, −0.47)	91.6%	< 0.05
12 months	4	−1.20	(−1.45, −0.96)	21.3%	< 0.05
Qigong	6	−1.10	(−1.32, −0.87)	48.3%	< 0.05
Baduanjin	8	−1.00	(−1.60, −0.40)	90.8%	< 0.05
Taiji	3	−1.51	(−3.08, 0.07)	95.5%	> 0.05
Comprehensive community-based intervention	10	−1.23	(−1.82, −0.64)	92.0%	< 0.05
Maintaining their original lifestyle	4	−1.16	(−1.65, −0.67)	57.3%	< 0.05
Walking	7	−0.60	(−0.95, 0.25)	61.8%	< 0.05

## Data Availability

All data supporting the findings of this study are derived from previously published articles, which are available in public databases as cited in the reference list. Extracted data and analysis files are available from the corresponding author upon reasonable request.

## Supporting Information

Supporting Information Additional supporting information can be found online in the Supporting Information section. The supporting information of this study include Appendix S1 and Tables S1–S9, which provide additional methodological details and extended results supporting the main findings of this systematic review and meta-analysis. Appendix S1 presents the specific literature retrieval strategy used for database searches. Figures S1 and S2 display the overall risk of bias assessment and methodological quality evaluation of the included studies. Figure S1: Bias risk proportion chart. Figure S2: Methodological quality assessment plot. Table S1 summarizes the basic characteristics of the included studies. Tables S2–S9 provide detailed subgroup analyses of the effects of traditional Chinese exercise on various metabolic outcomes in patients with prediabetes, including insulin resistance, glycated hemoglobin, postprandial blood glucose, and other secondary indicators. The PRISMA 2020 checklist is provided as supporting information to ensure adherence to reporting standards. All supporting information are available in the online version of this article.
