# Supplementary material for: Mind–Body Interventions for Prediabetes Management: Traditional Chinese Exercise and Its Dual Effects on Metabolic Control and Psychological Well-Being—A Systematic Review and Meta-Analysis
Source: J Diabetes Res. 2025 Nov 10;2025:8249301. doi: 10.1155/jdr/8249301 (PMC12623101; doi:10.1155/jdr/8249301)
Supplement: Supporting Information — Additional supporting information can be found online in the Supporting Information section. The supporting information of this study include Appendix S1 and Tables S1–S9, which provide additional methodological details and extended results supporting the main findings of this systematic review and meta-analysis. Appendix S1 presents the specific literature retrieval strategy used for database searches. Figures S1 and S2 display the overall risk of bias assessment and methodological quality evaluation of the included studies. Figure S1: Bias risk proportion chart. Figure S2: Methodological quality assessment plot. Table S1 summarizes the basic characteristics of the included studies. Tables S2–S9 provide detailed subgroup analyses of the effects of traditional Chinese exercise on various metabolic outcomes in patients with prediabetes, including insulin resistance, glycated hemoglobin, postprandial blood glucose, and other secondary indicators. The PRISMA 2020 checklist is provided as supporting information to ensure adherence to reporting standards. All supporting information are available in the online version of this article. [file 8249301.f1.docx]

**Appendix 1.** Specific retrieval strategy

**Embase**

Session Results

.......................................................

| No | Query | Results | Date |
| --- | --- | --- | --- |
| #3 | #1 AND #2 | 21 | 28-Aug-25 |
| #2 | 'impaired glucose tolerance'/exp OR 'state, prediabetic':ti,ab,kw OR 'chemical diabetes':ti,ab,kw OR 'diabetes mellitus, potential':ti,ab,kw OR 'diabetes, chemical':ti,ab,kw OR 'diabetes, latent':ti,ab,kw OR 'genetic prediabetes':ti,ab,kw OR 'glucose tolerance impairment':ti,ab,kw OR 'glucose tolerance, potentially impaired':ti,ab,kw OR 'impaired glucose tolerance, potential':ti,ab,kw OR 'latent diabetes':ti,ab,kw OR 'potential diabetes':ti,ab,kw OR 'potential glucose tolerance impairment':ti,ab,kw OR 'pre diabetes mellitus':ti,ab,kw OR prediabetes:ti,ab,kw OR 'prediabetes mellitus':ti,ab,kw OR 'prediabetic stage':ti,ab,kw OR 'prediabetic state':ti,ab,kw | 51408 | 28-Aug-25 |
| #1 | (('qigong'/exp OR 'qigong' OR 'qi gong':ti,ab,kw OR 'chi kung':ti,ab,kw OR chigung:ti,ab,kw) AND ('kung fu'/exp OR 'kung fu') OR kungfu:ti,ab,kw) AND ('tai chi'/exp OR 'tai chi') OR 'tai ji':ti,ab,kw OR 'chi, ai':ti,ab,kw OR 'ji quan, tai':ti,ab,kw OR 'quan, tai ji':ti,ab,kw OR taiji:ti,ab,kw OR 'tai chi':ti,ab,kw OR 'taiji quan':ti,ab,kw OR taijiquan:ti,ab,kw OR 'traditional exercise':ti,ab,kw OR wuqinxi:ti,ab,kw OR baduanjin:ti,ab,kw OR yijinjing:ti,ab,kw | 5612 | 28-Aug-25 |

.......................................................

**PubMed**

| Search number | Query | Results | Time | Date |
| --- | --- | --- | --- | --- |
| 5 | (((((((((((((((((((((Prediabetic State[MeSH Terms]) OR (Prediabetic State[Title/Abstract])) OR (impaired glucose tolerance[Title/Abstract])) OR (State, Prediabetic[Title/Abstract])) OR (Prediabetes[Title/Abstract])) OR (chemical diabetes[Title/Abstract])) OR (diabetes mellitus, potential[Title/Abstract])) OR (diabetes, chemical[Title/Abstract])) OR (diabetes, latent[Title/Abstract])) OR (genetic prediabetes[Title/Abstract])) OR (glucose tolerance impairment[Title/Abstract])) OR (glucose tolerance, potentially impaired[Title/Abstract])) OR (impaired glucose tolerance, potential[Title/Abstract])) OR (latent diabetes[Title/Abstract])) OR (potential diabetes[Title/Abstract])) OR (potential glucose tolerance impairment[Title/Abstract])) OR (pre diabetes mellitus[Title/Abstract])) OR (prediabetes[Title/Abstract])) OR (prediabetes mellitus[Title/Abstract])) OR (prediabetic stage[Title/Abstract])) OR (prediabetic state[Title/Abstract])) AND (((((((Qigong[MeSH Terms]) OR (Qigong[Title/Abstract])) OR (Qi Gong[Title/Abstract])) OR (Ch'i Kung[Title/Abstract])) OR (chi kung[Title/Abstract])) OR (chigung[Title/Abstract])) OR (((((((((((((((((((Tai Ji[MeSH Terms]) OR (Tai Ji[Title/Abstract])) OR (Martial Arts[Title/Abstract])) OR (kung fu[Title/Abstract])) OR (kungfu[Title/Abstract])) OR (Tai Chi[Title/Abstract])) OR (Tai-ji[Title/Abstract])) OR (Chi, Tai[Title/Abstract])) OR (Ji Quan, Tai[Title/Abstract])) OR (Quan, Tai Ji[Title/Abstract])) OR (Taiji[Title/Abstract])) OR (Taijiquan[Title/Abstract])) OR (T'ai Chi[Title/Abstract])) OR (Taiji quan[Title/Abstract])) OR (Taijiquan[Title/Abstract])) OR (Traditional exercise[Title/Abstract])) OR (Wuqinxi[Title/Abstract])) OR (Baduanjin[Title/Abstract])) OR (Yijinjing[Title/Abstract]))) | 15 | 4:31:43 | 2025/8/28 |
| 4 | ((((((Qigong[MeSH Terms]) OR (Qigong[Title/Abstract])) OR (Qi Gong[Title/Abstract])) OR (Ch'i Kung[Title/Abstract])) OR (chi kung[Title/Abstract])) OR (chigung[Title/Abstract])) OR (((((((((((((((((((Tai Ji[MeSH Terms]) OR (Tai Ji[Title/Abstract])) OR (Martial Arts[Title/Abstract])) OR (kung fu[Title/Abstract])) OR (kungfu[Title/Abstract])) OR (Tai Chi[Title/Abstract])) OR (Tai-ji[Title/Abstract])) OR (Chi, Tai[Title/Abstract])) OR (Ji Quan, Tai[Title/Abstract])) OR (Quan, Tai Ji[Title/Abstract])) OR (Taiji[Title/Abstract])) OR (Taijiquan[Title/Abstract])) OR (T'ai Chi[Title/Abstract])) OR (Taiji quan[Title/Abstract])) OR (Taijiquan[Title/Abstract])) OR (Traditional exercise[Title/Abstract])) OR (Wuqinxi[Title/Abstract])) OR (Baduanjin[Title/Abstract])) OR (Yijinjing[Title/Abstract])) | 6,121 | 4:31:30 | 2025/8/28 |
| 3 | ((((((((((((((((((((Prediabetic State[MeSH Terms]) OR (Prediabetic State[Title/Abstract])) OR (impaired glucose tolerance[Title/Abstract])) OR (State, Prediabetic[Title/Abstract])) OR (Prediabetes[Title/Abstract])) OR (chemical diabetes[Title/Abstract])) OR (diabetes mellitus, potential[Title/Abstract])) OR (diabetes, chemical[Title/Abstract])) OR (diabetes, latent[Title/Abstract])) OR (genetic prediabetes[Title/Abstract])) OR (glucose tolerance impairment[Title/Abstract])) OR (glucose tolerance, potentially impaired[Title/Abstract])) OR (impaired glucose tolerance, potential[Title/Abstract])) OR (latent diabetes[Title/Abstract])) OR (potential diabetes[Title/Abstract])) OR (potential glucose tolerance impairment[Title/Abstract])) OR (pre diabetes mellitus[Title/Abstract])) OR (prediabetes[Title/Abstract])) OR (prediabetes mellitus[Title/Abstract])) OR (prediabetic stage[Title/Abstract])) OR (prediabetic state[Title/Abstract]) | 29,793 | 4:31:19 | 2025/8/28 |
| 2 | ((((((((((((((((((Tai Ji[MeSH Terms]) OR (Tai Ji[Title/Abstract])) OR (Martial Arts[Title/Abstract])) OR (kung fu[Title/Abstract])) OR (kungfu[Title/Abstract])) OR (Tai Chi[Title/Abstract])) OR (Tai-ji[Title/Abstract])) OR (Chi, Tai[Title/Abstract])) OR (Ji Quan, Tai[Title/Abstract])) OR (Quan, Tai Ji[Title/Abstract])) OR (Taiji[Title/Abstract])) OR (Taijiquan[Title/Abstract])) OR (T'ai Chi[Title/Abstract])) OR (Taiji quan[Title/Abstract])) OR (Taijiquan[Title/Abstract])) OR (Traditional exercise[Title/Abstract])) OR (Wuqinxi[Title/Abstract])) OR (Baduanjin[Title/Abstract])) OR (Yijinjing[Title/Abstract]) | 5,327 | 4:31:07 | 2025/8/28 |
| 1 | (((((Qigong[MeSH Terms]) OR (Qigong[Title/Abstract])) OR (Qi Gong[Title/Abstract])) OR (Ch'i Kung[Title/Abstract])) OR (chi kung[Title/Abstract])) OR (chigung[Title/Abstract]) | 1,441 | 4:30:54 | 2025/8/28 |

**Web of Sicence**

(TI=((“Qigong”) OR (“Qi Gong”) OR (“Ch'i Kung”) OR (“chi kung”) OR (“chigung”) OR (“kung fu”) OR (“Kungfu”) OR (“Tai Ji”) OR (“Tai Chi”) OR (“Tai-ji”) OR (“Chi, Tai”) OR (“Ji Quan, Tai”) OR (“Quan, Tai Ji”) OR (“Taiji”) OR (“Taijiquan”) OR (“T'ai Chi”) OR (“Taiji quan”) OR (“Taijiquan”) OR (“Traditional exercise”) OR (“Wuqinxi”) OR (“Baduanjin”) OR (“Yijinjing”)) OR AB=((“Qigong”) OR (“Qi Gong”) OR (“Ch'i Kung”) OR (“chi kung”) OR (“chigung”) OR (“kung fu”) OR (“Kungfu”) OR (“Tai Ji”) OR (“Tai Chi”) OR (“Tai-ji”) OR (“Chi, Tai”) OR (“Ji Quan, Tai”) OR (“Quan, Tai Ji”) OR (“Taiji”) OR (“Taijiquan”) OR (“T'ai Chi”) OR (“Taiji quan”) OR (“Taijiquan”) OR (“Traditional exercise”) OR (“Wuqinxi”) OR (“Baduanjin”) OR (“Yijinjing”)) OR AK=((“Qigong”) OR (“Qi Gong”) OR (“Ch'i Kung”) OR (“chi kung”) OR (“chigung”) OR (“kung fu”) OR (“Kungfu”) OR (“Tai Ji”) OR (“Tai Chi”) OR (“Tai-ji”) OR (“Chi, Tai”) OR (“Ji Quan, Tai”) OR (“Quan, Tai Ji”) OR (“Taiji”) OR (“Taijiquan”) OR (“T'ai Chi”) OR (“Taiji quan”) OR (“Taijiquan”) OR (“Traditional exercise”) OR (“Wuqinxi”) OR (“Baduanjin”) OR (“Yijinjing”))) AND (TI=((“Prediabetic State”) OR (“impaired glucose tolerance”) OR (“State, Prediabetic”) OR (“Prediabetes”) OR (“chemical diabetes”) OR (“diabetes mellitus, potential”) OR (“diabetes, chemical”) OR (“diabetes, latent”) OR (“genetic prediabetes”) OR (“glucose tolerance impairment”) OR (“glucose tolerance, potentially impaired”) OR (“impaired glucose tolerance, potential”) OR (“latent diabetes”) OR (“potential diabetes”) OR (“potential glucose tolerance impairment”) OR (“pre diabetes mellitus”) OR (“prediabetes”) OR (“prediabetes mellitus”) OR (“prediabetic stage”) OR (“prediabetic state”)) OR AB=((“Prediabetic State”) OR (“impaired glucose tolerance”) OR (“State, Prediabetic”) OR (“Prediabetes”) OR (“chemical diabetes”) OR (“diabetes mellitus, potential”) OR (“diabetes, chemical”) OR (“diabetes, latent”) OR (“genetic prediabetes”) OR (“glucose tolerance impairment”) OR (“glucose tolerance, potentially impaired”) OR (“impaired glucose tolerance, potential”) OR (“latent diabetes”) OR (“potential diabetes”) OR (“potential glucose tolerance impairment”) OR (“pre diabetes mellitus”) OR (“prediabetes”) OR (“prediabetes mellitus”) OR (“prediabetic stage”) OR (“prediabetic state”)) OR AK=((“Prediabetic State”) OR (“impaired glucose tolerance”) OR (“State, Prediabetic”) OR (“Prediabetes”) OR (“chemical diabetes”) OR (“diabetes mellitus, potential”) OR (“diabetes, chemical”) OR (“diabetes, latent”) OR (“genetic prediabetes”) OR (“glucose tolerance impairment”) OR (“glucose tolerance, potentially impaired”) OR (“impaired glucose tolerance, potential”) OR (“latent diabetes”) OR (“potential diabetes”) OR (“potential glucose tolerance impairment”) OR (“pre diabetes mellitus”) OR (“prediabetes”) OR (“prediabetes mellitus”) OR (“prediabetic stage”) OR (“prediabetic state”)))

14 hits 2025/8/28

**Cochrane Library**

Search Name:

Date Run: 28/08/2025 11:06:01

Comment:

ID Search Hits

#1 MeSH descriptor: [Qigong] explode all trees 180

#2 (Qi Gong):ti,ab,kw OR (Ch'i Kung):ti,ab,kw OR (chi kung):ti,ab,kw OR (chigung):ti,ab,kw 106

#3 MeSH descriptor: [Martial Arts] explode all trees 834

#4 (kung fu):ti,ab,kw OR (kungfu):ti,ab,kw 28

#5 MeSH descriptor: [Tai Ji] explode all trees 603

#6 (Tai Chi):ti,ab,kw OR (Tai-ji):ti,ab,kw OR (Chi, Tai):ti,ab,kw OR (Ji Quan, Tai):ti,ab,kw OR (Quan, Tai Ji):ti,ab,kw 2098

#7 (Taiji):ti,ab,kw OR (Taijiquan):ti,ab,kw OR (T'ai Chi):ti,ab,kw OR (Taiji quan):ti,ab,kw OR (Taijiquan):ti,ab,kw 245

#8 (Traditional exercise):ti,ab,kw OR (Wuqinxi):ti,ab,kw OR (Baduanjin):ti,ab,kw OR (Yijinjing):ti,ab,kw 5430

#9 MeSH descriptor: [Prediabetic State] explode all trees 1858

#10 (impaired glucose tolerance):ti,ab,kw OR (State, Prediabetic):ti,ab,kw OR (Prediabetes):ti,ab,kw OR (chemical diabetes):ti,ab,kw OR (diabetes mellitus, potential):ti,ab,kw 14282

#11 (diabetes, chemical):ti,ab,kw OR (diabetes, latent):ti,ab,kw OR (genetic prediabetes):ti,ab,kw OR (glucose tolerance impairment):ti,ab,kw OR (glucose tolerance, potentially impaired):ti,ab,kw 1278

#12 (impaired glucose tolerance, potential):ti,ab,kw OR (latent diabetes):ti,ab,kw OR (potential diabetes):ti,ab,kw OR (potential glucose tolerance impairment):ti,ab,kw OR (pre diabetes mellitus):ti,ab,kw 16337

#13 (prediabetes):ti,ab,kw OR (prediabetes mellitus):ti,ab,kw OR (prediabetic stage):ti,ab,kw OR (prediabetic state):ti,ab,kw 4115

#14 #1 or #2 or #3 or #4 or #5 or #6 or #7 or #8 7863

#15 #9 or #10 or #11 or #12 or #13 22474

#16 #14 and #15 115

**Chinese retrieval strategy**

**VIP**

糖尿病前期+prediabetes+prediabetic state+耐量异常+糖耐量低减+糖耐量减低+糖耐量降低+葡萄糖耐量受损+糖调节异常+糖耐量受损+糖调节受损+糖耐量减退+葡萄糖耐量减低+糖耐量异常+葡萄糖耐量异常

八段锦+太极+太极拳+易筋经+五禽戏+功夫+气功+中国传统运动+中国传统训练+传统运动+传统训练+中医运动+中医功法

**Wanfang**

糖尿病前期 or耐量异常 or 糖耐量低减 or 糖耐量减低 or 糖耐量降低 or糖调节异常 or 糖耐量受损 or 糖调节受损 or 糖耐量减退

八段锦 or 太极 or 太极拳 or 易筋经 or 五禽戏 or 功夫 or 气功 or 中国传统运动 or 中国传统训练 or 传统运动 or 传统训练 or 中医运动 or 中医功法

**China National Knowledge Infrastructure (CNKI)**

TKA=('糖尿病前期'+'prediabetes'+'prediabetic state'+'耐量异常'+'糖耐量低减'+'糖耐量减低'+'糖耐量降低'+'葡萄糖耐量受损'+'糖调节异常'+'糖耐量受损'+'糖调节受损'+'糖耐量减退'+'葡萄糖耐量减低'+'糖耐量异常'+'葡萄糖耐量异常')

AND TKA=('八段锦'+'太极'+'太极拳'+'易筋经'+'五禽戏'+'功夫'+'气功'+'中国传统运动'+'中国传统训练'+'传统运动'+'传统训练'+'中医运动'+'中医功法')

**CBM**

( "糖尿病前期"[全部字段:智能] OR "耐量异常"[全部字段:智能] OR "糖耐量低减"[全部字段:智能] OR "糖耐量减低"[全部字段:智能] OR "糖耐量降低"[全部字段:智能] OR "糖调节异常"[全部字段:智能] OR "糖耐量受损"[全部字段:智能] OR "糖调节受损"[全部字段:智能] OR "糖耐量减退"[全部字段:智能]) AND( "八段锦"[全部字段:智能] OR "太极"[全部字段:智能] OR "太极拳"[全部字段:智能] OR "易筋经"[全部字段:智能] OR "五禽戏"[全部字段:智能] OR "功夫"[全部字段:智能] OR "气功"[全部字段:智能] OR "中国传统运动"[全部字段:智能] OR "中国传统训练"[全部字段:智能] OR "传统运动"[全部字段:智能] OR "传统训练"[全部字段:智能] OR "中医运动"[全部字段:智能] OR "中医功法"[全部字段:智能])

**Supplementary Figure S1.** Bias risk proportion chart.

**Supplementary Figure S2.** Methodological quality assessment plot.

**Supplementary Table S1** Basic characteristics of the included literature

| Author, Year | Country | Type of work | Treatment | | Sample Size* (E/C) | Stage Classification | FollowUp time | Outcome indicators |
| --- | --- | --- | --- | --- | --- | --- | --- | --- |
|  |  |  | Experiment group | Control group |  |  |  |  |
| ChaoWang  2010 | China | RCT | Yijinjing | comprehensive community-based interventions | 30 | 30 | 3 mon | ①⑮⑰ |
| YanmingWu  2011 | China | RCT | Baduanjin | 1.maintain their  regular lifestyle  2.comprehensive community-based interventions | 56/59.60 | Prediabetes | 6 mon | ⑯ |
| HonanaSong  2013 | China | RCT | TaiChi | comprehensive community-based interventions | 40/40 | Prediabetes | 3 mon | ①②③④⑤⑥ |
| ChunpingFang  2014 | China | RCT | Baduanjin | 1.comprehensive community-based interventions  2.walking | 28/30.28 | Prediabetes | 12 mon | ⑤⑥⑦⑧⑨⑩ |
| ChunpingFang  2014 | China | RCT | Baduanjin | 1.comprehensive community-based interventions  2.walking | 28/30.28 | Prediabetes | 12 mon | ①②③⑪⑫⑬⑭ |
| YongyiLu  2014 | China | RCT | Yijingjin | comprehensive community-based interventions | 60/60 | Prediabetes | 12 mon | ①③⑯⑰ |
| YunchuanWu  2015 | China | RCT | Shaolin Qigong | 1.maintain their regular lifestyle  2.walking | 20/20.20 | Prediabetes | 3 mon | ①②③⑮⑱ |
| KunZhao  2016 | China | RCT | Baduanjin | 1.comprehensive community-based interventions  2.walking | 20/30.30 | Prediabetes | 12 mon | ⑤⑥⑦⑧⑨⑩ |
| YunchuanWu  2016 | China | RCT | Baduanjin | 1.comprehensive community-based interventions  2.walking | 60/20.20 | Prediabetes | 6 mon | ①②③⑱⑲ |
| PengLi  2017 | China | RCT | Shaolin Qigong | 1.maintain their regular lifestyle  2.walking | 40/10.10 | Prediabetes | 6 mon | ①②③ |
| YuehuiShang  2017 | China | RCT | Baduanjin | walking | 43/43 | Prediabetes | 6 mon | ①②⑪⑫⑬⑭⑲⑳ |
| TongZhang  2018 | China | RCT | Shaolin Qigong | 1.maintain their regular lifestyle  2.walking | 30/31.31 | Prediabetes | 6 mon | ④ |
| JunxiaoZhu  2019 | China | RCT | Chinese exercise | comprehensive community-based interventions | 28/28 | Prediabetes | 12 mon | ①③⑲⑳⑰ |
| WenhaoLi  2019 | China | RCT | Baduanjin | comprehensive community-based interventions | 49/47 | Prediabetes | 9 mon | ①④⑮ |
| WenhaoLi  2019 | China | RCT | TaiChi | comprehensive community-based interventions | 43/44 | Prediabetes | 6 mon | ① |
| YingShen  2020 | China | RCT | Baduanjin | walking | 52/51 | Prediabetes | 6 mon | ①②⑪⑫⑬⑭⑲⑳ |
| XiaojunMa  2022 | China | RCT | Baduanjin | maintain their regular lifestyle | 34/32 | Prediabetes | 12 mon | ①②③⑪⑫⑬⑭⑯ |
| ShujuanHu  2022 | China | RCT | TaiChi | 1.maintain their regular lifestyle  2.healthy normal person | 19/19.10 | Prediabetes | 3 mon | ①②③④⑪⑫⑬⑭⑮⑲⑳ |
| PingXu  2022 | China | RCT | Baduanjin | maintain their regular lifestyle | 20/20 | Prediabetes | 3 mon | ①②⑪⑫⑬⑭ |
| QinLi  2022 | China | RCT | Baduanjin | walking | 39/37 | Prediabetes | 6 mon | ①②③④⑪⑫⑬⑭⑮⑱⑲⑳ |
| ZhenghongYe  2025 | China | RCT | WuqinXi | walking | 30/30 | Prediabetes | 1 mon | ①②③④⑮ |
| YuhanZeng  2025 | China | RCT | WuqinXi | walking | 30/30 | Prediabetes | 1 mon | ⑪⑫⑬⑭ |

Note: ①FBG; ②2hBG; ③HbA1c; ④ HOMA-IR; ⑤Depression factor score; ⑥ Anxiety; ⑦ Somatization factor score; ⑧Interpersonal relationship sensitivity factor score; ⑨Paranoia factor score; ⑩Psychotic factor score; ⑪ LDL-C; ⑫ HDL-C; ⑬ TC; ⑭ TG; ⑮ FINS; ⑯ Transition to T2DM; ⑰ OGTT; ⑱SF-36; ⑲ BMI; ⑳ WHR. Comprehensive community-based interventions include health education and dietary education. The intervention method for participating in walking exercises is to walk at a moderate pace (80-100 steps/min) twice a day, 30 minutes each time, exercising 5 days or more per week, or walking daily before breakfast and after dinner, with each walk for about 0.5 hours, continuously walking for 5 days a week, maintaining an appropriate walking speed with the walking speed calculated as pulse rate (times/min) = [170-age].

*Since some studies have multiple control groups, the sample size of the control group has multiple sets of numbers, which is explained.

**Supplementary Table S2.** Effects of traditional Chinese medicine exercise on insulin resistance index in patients with prediabetes across different subgroups.

| Subgroup factors | K (the  Number  of studies) | SMD  (the pooled effect size) | 95%CI | I^2^ value | P value |
| --- | --- | --- | --- | --- | --- |
| 3 mon | 2 | -2.36 | (-5.77, 1.05) | 97.8% | ＞ 0.05 |
| 6 mon | 2 | -1.27 | (-2.63, 0.09) | 92.4% | ＞ 0.05 |
| Qigong | 2 | -0.54 | (-0.90, -0.18) | 0.0% | ＜ 0.05 |
| Baduanjin | 2 | -1.61 | (-2.26, -0.95) | 71.1% | ＜ 0.05 |
| Taiji | 2 | -2.36 | (-5.77, 1.05) | 93.9% | ＞ 0.05 |
| Comprehensive  community-based intervention | 2 | -2.65 | (-5.46, 0.17) | 97.5% | ＞ 0.05 |
| Walking | 3 | -0.90 | (-1.92, 0.13) | 95.1% | ＞ 0.05 |
| Maintaining  the original lifestyle | 2 | -0.84 | (-1.25, -0.43) | 0.0% | ＜ 0.05 |

**Supplementary Table S3** Effects of traditional Chinese medicine exercise on glycated hemoglobin in patients with prediabetes across different subgroups.

| Subgroup factors | K (the  Number  of studies) | SMD  (the pooled effect size) | 95%CI | I^2^ value | P value |
| --- | --- | --- | --- | --- | --- |
| 3 mon | 6 | -0.30 | (-0.53, -0.06) | 0.0% | ＜ 0.05 |
| 6 mon | 4 | -0.57 | (-0.85, -0.29) | 0.0% | ＜ 0.05 |
| 12 mon | 4 | -1.16 | (-2.50, 0.18) | 96.2% | ＞ 0.05 |
| Qigong | 5 | -0.88 | (-2.15, 0.39) | 95.7% | ＞ 0.05 |
| Baduanjin | 4 | -0.55 | (-0.81, -0.29) | 42.0% | ＜ 0.05 |
| Taiji | 2 | -0.50 | (-1.04, 0.04) | 90.7% | ＞ 0.05 |
| Comprehensive  community-based  intervention | 6 | -0.91 | (-1.89, 0.07) | 94.5% | ＞ 0.05 |
| Walking | 6 | -0.21 | (-0.44, 0.02) | 0.0% | ＞ 0.05 |
| Maintaining the  original lifestyle | 3 | -0.80 | (-1.14, -0.45) | 0.0% | ＜ 0.05 |

**Supplementary Table S4** Effects of traditional Chinese medicine exercise on 2-hour post-meal blood glucose in patients with prediabetes across different subgroups.

| Subgroup factors | K (the  Number  of studies) | SMD  (the pooled effect size) | 95%CI | I^2^ value | P value |
| --- | --- | --- | --- | --- | --- |
| 3 mon | 7 | -0.72 | (-0.95, -0.49) | 0.0% | ＜ 0.05 |
| 6 mon | 6 | -0.48 | (-0.68, -0.28) | 0.0% | ＜ 0.05 |
| 12 mon | 2 | -0.63 | (-1.61, 0.35) | 86.0% | ＞ 0.05 |
| Qigong | 3 | -0.75 | (-1.13, -0.38) | 0.0% | ＜ 0.05 |
| Baduanjin | 7 | -0.50 | (-0.69, -0.32) | 46.6% | ＜ 0.05 |
| Taiji | 2 | -0.93 | (-1.31, -0.55) | 0.0% | ＜ 0.05 |
| Comprehensive community-based  intervention | 6 | -0.75 | (-0.97, -0.53) | 0.0% | ＜ 0.05 |
| Walking | 7 | -0.22 | (-0.42, -0.02) | 34.3% | ＜ 0.05 |
| Maintaining the  original lifestyle | 3 | -1.20 | (-1.56, -0.84) | 0.0% | ＜ 0.05 |

**Supplementary Table S5** Summary of Secondary Outcome Indicators Analysis.

| Outcome indicators name | N (number of articles) | SMD/RR* | 95%CI | I2 | P (statistic difference) |
| --- | --- | --- | --- | --- | --- |
| Depression factor score | 3 | -0.49 | (-0.77, -0.20) | 30.4% | ＜ 0.05 |
| Anxiety factor score | 3 | -0.94 | (-1.38, -0.51) | 54.2% | ＜ 0.05 |
| Somatization factor score | 2 | -0.40 | (-0.77, -0.03) | 0.0% | ＜ 0.05 |
| Interpersonal sensitivity factor score | 2 | -0.27 | (-0.64, 0.10) | 0.0% | ＞ 0.05 |
| Paranoia factor score | 2 | -0.29 | (-0.66, 0.07) | 0.0% | ＞ 0.05 |
| Psychotic factor score | 2 | -0.26 | (-0.62, 0.11) | 0.0% | ＞ 0.05 |
| LDL-C | 8 | -0.47 | (-0.96, 0.01) | 86.4% | ＞ 0.05 |
| HDL-C | 8 | 0.24 | (-0.17, 0.64) | 81.1% | ＞ 0.05 |
| TC | 8 | -0.46 | (-0.75, -0.18) | 60.8% | ＜ 0.05 |
| TG | 8 | -0.70 | (-1.20, -0.19) | 87.0% | ＜ 0.05 |
| FINS | 6 | -0.14 | (-0.35, 0.06) | 0.0% | ＞ 0.05 |
| SF-36 | 3 | 1.03 | (0.60, 1.46) | 36.8% | ＞ 0.05 |
| BMI | 6 | -0.77 | (-1.58, 0.05) | 92.9% | ＞ 0.05 |
| WHR | 5 | -0.94 | (-1.48 ,-0.40) | 82.4% | ＜ 0.05 |
| the occurrence of events converting to T2DM | 3 | 0.24 | (0.10, 0.60) | 0.0% | ＜ 0.05 |

* Only the occurrence of events converting to T2DM was RR, and SMD was used in the rest of the studies

**Supplementary Table S6** Summary of Subgroup Analysis of Secondary Outcome Indicators Based on Intervention Duration.

| Outcome indicators name | Subgroup factors | | N (number of articles) | SMD | 95%CI | I2 | P (statistic difference) |
| --- | --- | --- | --- | --- | --- | --- | --- |
| Depression factor score | Intervention duration | 12mon | 2 | -0.64 | (-1.01, -0.27) | 18.0% | ＜ 0.05 |
| Anxiety factor score | Intervention duration | 12mon | 2 | -0.73 | (-1.10, -0.36) | 0.0% | ＜ 0.05 |
| LDL-C | Intervention duration | 3mon | 3 | -0.64 | (-1.45, 0.17) | 81.3% | ＞ 0.05 |
|  |  | 6mon | 4 | -0.58 | (-1.13, -0.03) | 83.4% | ＜ 0.05 |
|  |  | 12mon | 2 | -0.36 | (-1.41, 0.69) | 88.1% | ＞ 0.05 |
| HDL-C | Intervention duration | 3mon | 3 | 0.10 | (-0.22, 0.43) | 0.0% | ＞ 0.05 |
|  |  | 6mon | 4 | 0.19 | (-0.02, 0.41) | 16.1% | ＞ 0.05 |
|  |  | 12mon | 2 | 1.01 | (-0.63, 2.64) | 94.4% | ＞ 0.05 |
| TC | Intervention duration | 3mon | 3 | -0.44 | (-0.77, -0.11) | 47.3% | ＜ 0.05 |
|  |  | 6mon | 4 | -0.44 | (-0.88, -0.01) | 74.6% | ＜ 0.05 |
|  |  | 12mon | 2 | -0.53 | (-0.89, -0.17) | 0.0% | ＜ 0.05 |
| TG | Intervention duration | 3mon | 3 | -0.96 | (-2.07, 0.15) | 89.2% | ＞ 0.05 |
|  |  | 6mon | 4 | -0.62 | (-1.27, 0.04) | 88.1% | ＞ 0.05 |
|  |  | 12mon | 2 | -0.42 | (-1.58, 0.73) | 89.9% | ＞ 0.05 |
| FINS | Intervention duration | 3mon | 4 | -0.46 | (-0.72, -0.20) | 43.2% | ＜ 0.05 |
|  |  | 6mon | 2 | -0.75 | (-1.75, 0.25) | 90.1% | ＞ 0.05 |
| SF-36 | Intervention duration | 6mon | 2 | 1.23 | (0.83, 1.63) | 0.0% | ＞ 0.05 |
| BMI | Intervention duration | 3mon | 2 | -0.14 | (-0.58, 0.31) | 0.0% | ＞ 0.05 |
|  |  | 6mon | 4 | -1.08 | (-2.17, 0.01) | 94.5% | ＞ 0.05 |
| WHR | Intervention duration | 6mon | 3 | -0.89 | (-1.68, -0.11) | 89.2% | ＜ 0.05 |

**Supplementary Table S7** Summary of Subgroup Analysis of Secondary Outcome Indicators Based on Intervention Group Types.

| Names of outcome indicators | Subgroup factors | | N (number of articles) | SMD | 95%CI | I2 | P (statistically significant difference) |
| --- | --- | --- | --- | --- | --- | --- | --- |
| Depressive factor score | Interventions | Baduanjin | 2 | -0.64 | (-1.01, -0.27) | 18.0% | ＜ 0.05 |
|  |  | Taiji | 1 | -0.27 | (-0.71, 0.17) | / | / |
| FINS | Interventions | Qigong | 3 | -0.11 | (-0.42, 0.20) | 0.0% | ＞ 0.05 |
|  |  | Taiji | 1 | -0.44 | (-1.08, 0.21) | / | / |
|  |  | Baduanjin | 2 | -0.11 | (-0.41, 0.19) | 0.0% | ＞ 0.05 |
| Anxiety  factor score | Interventions | Baduanjin | 2 | -0.73 | (-1.10, -0.36) | 0.0% | ＜ 0.05 |
|  |  | Taiji | 1 | -1.34 | (-1.83, -0.86) | / | / |
|  |  | Walking | 2 | -0.25 | (-0.62, 0.11) | 55.7% | ＞ 0.05 |
| BMI | Interventions | Qigong | 1 | -0.00 | (-0.53, 0.52) | / | / |
|  |  | Taiji | 1 | -0.26 | (-0.90, 0.38) | / | / |
|  |  | Baduanjin | 4 | -1.08 | (-2.71, 0.01) | 94.5% | ＞ 0.05 |
| SF-36 | Interventions | Baduanjin | 2 | 1.23 | (0.83, 1.63) | 0.0% | ＞ 0.05 |
|  |  | Qigong | 1 | 0.58 | (-0.05, 1.22) | / | / |
| HDL-C | Interventions | Baduanjin | 6 | 0.37 | (-0.13, 0.87) | 84.4% | ＞ 0.05 |
|  |  | Taiji | 1 | -0.08 | (-0.72, 0.55) | / | / |
|  |  | Qigong | 1 | -0.27 | (-0.78, 0.24) | / | / |
| TG | Interventions | Baduanjin | 6 | -0.80 | (-1.47, -0.13) | 90.6% | ＜ 0.05 |
|  |  | Taiji | 1 | -0.34 | (-0.98, 0.30) | / | / |
|  |  | Qigong | 1 | -0.50 | (-1.01, 0.01) | / | / |
| TC | Interventions | Baduanjin | 6 | -0.56 | (-0.90, -0.22) | 86.1% | < 0.05 |
|  |  | Taiji | 1 | -0.27 | (-0.91, 0.37) | / | / |
|  |  | Qigong | 1 | -0.05 | (-0.56, 0.46) | / | / |
| WHR | Interventions | Qigong | 1 | -0.59 | (-1.12, -0.05) | / | / |
|  |  | Taiji | 1 | -1.53 | (-2.25, -0.80) | / | / |
|  |  | Baduanjin | 3 | -0.89 | (-1.68, -0.11) | 89.2% | ＜ 0.05 |

**Supplementary Table S8** Summary of Subgroup Analysis of Secondary Outcome Indicators Based on Control Group Types.

| Names of outcome indicators | Subgroup factors | | N (number of articles) | SMD | 95%CI | I2 | P (statistically significant difference) |
| --- | --- | --- | --- | --- | --- | --- | --- |
| Depressive factor score | Interventions | Comprehensive community-based intervention | 3 | -0.49 | (-0.77, -0.20) | 30.4% | ＜ 0.05 |
|  |  | Walking | 2 | -0.31 | (-0.67, 0.05) | 0.0% | ＞ 0.05 |
| FINS | Interventions | Maintaining the  original lifestyle | 2 | -0.36 | (-0.80, 0.09) | 0.0% | ＞ 0.05 |
|  |  | Comprehensive community-based intervention | 2 | -0.05 | (-0.44, 0.15) | 0.0% | ＞ 0.05 |
|  |  | Walking | 3 | -0.15 | (-0.44, 0.15) | 0.0% | ＞ 0.05 |
| Anxiety factor score | Interventions | Comprehensive community-based intervention | 3 | -0.79 | (-1.51, -0.08) | 83.1% | ＜ 0.05 |
|  |  | Walking | 2 | -0.99 | (-1.40, -0.59) | 11.2% | ＜ 0.05 |
| Psychotic factor score | Interventions | Comprehensive community-based intervention | 2 | -0.29 | (-0.65, 0.08) | 0.0% | ＞ 0.05 |
|  |  | Walking | 2 | -0.23 | (-0.60, 0.13) | 0.0% | ＞ 0.05 |
| Paranoia factor score | Interventions | Comprehensive community-based intervention | 2 | -0.36 | (-0.73, 0.00) | 0.0% | ＞ 0.05 |
|  |  | Walking | 2 | -0.21 | (-0.58, 0.15) | 0.0% | ＞ 0.05 |
| Interpersonal sensitivity factor score | Interventions | Comprehensive community-based intervention | 2 | -0.32 | (-0.68, 0.04) | 0.0% | ＞ 0.05 |
|  |  | Walking | 2 | -0.21 | (-0.58, 0.15) | 48.0% | ＞ 0.05 |
| Somatization factor score | Interventions | Comprehensive community-based intervention | 2 | -0.57 | (-0.94, -0.20) | 0.0% | ＜ 0.05 |
|  |  | Walking | 2 | -0.25 | (-0.62, 0.11) | 55.7% | ＞ 0.05 |
| BMI | Interventions | Comprehensive community-based intervention | 2 | 0.01 | (-0.39, 0.41) | 0.0% | ＞ 0.05 |
|  |  | Walking | 4 | -1.09 | (-2.17, -0.01) | 94.4% | ＜ 0.05 |
|  |  | Maintaining the original lifestyle | 1 | -0.26 | (-0.90, 0.38) | / | / |
| SF-36 | Interventions | Comprehensive community-based intervention | 1 | 1.49 | (0.79, 2.20) | / | / |
|  |  | Walking | 2 | 1.12 | (0.73, 1.51) | 36.0% | ＞ 0.05 |
|  |  | Maintaining the original lifestyle | 1 | 0.58 | (-0.05, 1.22) | / | / |
| HDL-C | Interventions | Comprehensive community-based intervention | 3 | 0.21 | (-0.07, 0.48) | 0.0% | ＞ 0.05 |
|  |  | Walking | 4 | -0.04 | (-0.27, 0.20) | 0.0% | ＞ 0.05 |
|  |  | Maintaining the original lifestyle | 2 | 0.89 | (-1.00, 2.78) | 94.8% | ＞ 0.05 |
| TG | Interventions | Comprehensive community-based intervention | 3 | -0.96 | (-1.97, 0.05) | 90.2% | ＞ 0.05 |
|  |  | Walking | 4 | -0.31 | (-1.27, 0.65) | 93.3% | ＞ 0.05 |
|  |  | Maintaining the original lifestyle | 2 | -0.69 | (-1.37, -0.01) | 63.7% | ＜ 0.05 |
| TC | Interventions | Comprehensive community-based intervention | 3 | -0.51 | (-0.79, -0.23) | 25.4% | ＜ 0.05 |
|  |  | Walking | 4 | -0.37 | (-0.88, 0.14) | 77.7% | ＞ 0.05 |
|  |  | Maintaining the  original lifestyle | 2 | -0.52 | (-0.91, -0.12) | 0.0% | ＜ 0.05 |
| WHR | Interventions | Comprehensive community-based intervention | 2 | -1.15 | (-2.24, -0.06) | 89.7% | ＜ 0.05 |
|  |  | Maintaining the original lifestyle | 1 | -1.53 | (-2.25, -0.80) | / | / |
|  |  | Walking | 2 | -0.49 | (-0.81, -0.18) | 0.0% | ＜ 0.05 |

**Supplementary Table S9** Egger's analysis results of FBG.

| **Outcome indicators** | **Item** | **Effect size** | **Standard error** | **95%CI** | **t-value** | **p** |
| --- | --- | --- | --- | --- | --- | --- |
| FBG 3mon | Slope | -1.182873 | 1.114815 | -3.910727, 1.544981 | -1.06 | 0.329 |
|  | Bias | 1.496822 | 3.796898 | -7.793851, 10.7875 | 0.39 | 0.707 |
| FBG 6mon | Slope | -0.2840792 | 1.605936 | -4.213664, 3.645506 | -0.18 | 0.865 |
|  | Bias | -3.012354 | 6.113323 | -17.97112, 11.94641 | -0.49 | 0.640 |
| FBG 12mon | Slope | -2.127622 | 0.9743396 | -6.319867, 2.064623 | -2.18 | 0.161 |
|  | Bias | 3.687149 | 3.848159 | -12.87014, 20.24444 | 0.96 | 0.439 |

**PRISMA_2020_checklist**

| **Section and Topic** | **Item #** | **Checklist item** | **Location where item is reported** |
| --- | --- | --- | --- |
| **TITLE** | | |  |
| Title | 1 | Identify the report as a systematic review. | Page1, line 2-3 |
| **ABSTRACT** | | |  |
| Abstract | 2 | See the PRISMA 2020 for Abstracts checklist. | Pages 2-3, line 43-60 |
| **INTRODUCTION** | | |  |
| Rationale | 3 | Describe the rationale for the review in the context of existing knowledge. | Pages 3-4, line 63-106 |
| Objectives | 4 | Provide an explicit statement of the objective(s) or question(s) the review addresses. | Page 4,line 107-110 |
| **METHODS** | | |  |
| Eligibility criteria | 5 | Specify the inclusion and exclusion criteria for the review and how studies were grouped for the syntheses. | Pages 5, line 118-142 |
| Information sources | 6 | Specify all databases, registers, websites, organisations, reference lists and other sources searched or consulted to identify studies. Specify the date when each source was last searched or consulted. | Page 6, line 144-149 |
| Search strategy | 7 | Present the full search strategies for all databases, registers and websites, including any filters and limits used. | Page 6, line 144-149 & Appendix 1 |
| Selection process | 8 | Specify the methods used to decide whether a study met the inclusion criteria of the review, including how many reviewers screened each record and each report retrieved, whether they worked independently, and if applicable, details of automation tools used in the process. | Page 6 line 151-158 |
| Data collection process | 9 | Specify the methods used to collect data from reports, including how many reviewers collected data from each report, whether they worked independently, any processes for obtaining or confirming data from study investigators, and if applicable, details of automation tools used in the process. | Page 6 line 151-158 |
| Data items | 10a | List and define all outcomes for which data were sought. Specify whether all results that were compatible with each outcome domain in each study were sought (e.g. for all measures, time points, analyses), and if not, the methods used to decide which results to collect. | Page 6 line 151-158 |
|  | 10b | List and define all other variables for which data were sought (e.g. participant and intervention characteristics, funding sources). Describe any assumptions made about any missing or unclear information. | Page 6 line 151-158 |
| Study risk of bias assessment | 11 | Specify the methods used to assess risk of bias in the included studies, including details of the tool(s) used, how many reviewers assessed each study and whether they worked independently, and if applicable, details of automation tools used in the process. | Page 6, line 160-168 |
| Effect measures | 12 | Specify for each outcome the effect measure(s) (e.g. risk ratio, mean difference) used in the synthesis or presentation of results. | Pages 6-7, line 170-183 |
| Synthesis methods | 13a | Describe the processes used to decide which studies were eligible for each synthesis (e.g. tabulating the study intervention characteristics and comparing against the planned groups for each synthesis (item #5)). | Pages 6-7, line 170-183 |
|  | 13b | Describe any methods required to prepare the data for presentation or synthesis, such as handling of missing summary statistics, or data conversions. | Pages 7-8, line 194-201 |
|  | 13c | Describe any methods used to tabulate or visually display results of individual studies and syntheses. | Pages 6-7, line 170-183 |
|  | 13d | Describe any methods used to synthesize results and provide a rationale for the choice(s). If meta-analysis was performed, describe the model(s), method(s) to identify the presence and extent of statistical heterogeneity, and software package(s) used. | Pages 6-7, line 170-183 |
|  | 13e | Describe any methods used to explore possible causes of heterogeneity among study results (e.g. subgroup analysis, meta-regression). | Page 7, line 178-180 |
|  | 13f | Describe any sensitivity analyses conducted to assess robustness of the synthesized results. | Page 7, line 181 |
| Reporting bias assessment | 14 | Describe any methods used to assess risk of bias due to missing results in a synthesis (arising from reporting biases). | Page 7, line 181-183 |
| Certainty assessment | 15 | Describe any methods used to assess certainty (or confidence) in the body of evidence for an outcome. | Pagess 7-8, line 194-201 |
| **RESULTS** | | |  |
| Study selection | 16a | Describe the results of the search and selection process, from the number of records identified in the search to the number of studies included in the review, ideally using a flow diagram. | Page 8, line 204-208 & Figure 1 |
|  | 16b | Cite studies that might appear to meet the inclusion criteria, but which were excluded, and explain why they were excluded. | Page 8, line 204-208 & Figure 1 |
| Study characteristics | 17 | Cite each included study and present its characteristics. | Page 8, line 209-214& Table S1 |
| Risk of bias in studies | 18 | Present assessments of risk of bias for each included study. | Page 8, line 216-227 & Figure S1, S2 |
| Results of individual studies | 19 | For all outcomes, present, for each study: (a) summary statistics for each group (where appropriate) and (b) an effect estimate and its precision (e.g. confidence/credible interval), ideally using structured tables or plots. | Figures 2-6, Tables S1-S9 |
| Results of syntheses | 20a | For each synthesis, briefly summarise the characteristics and risk of bias among contributing studies. | The article has been supplemented |
|  | 20b | Present results of all statistical syntheses conducted. If meta-analysis was done, present for each the summary estimate and its precision (e.g. confidence/credible interval) and measures of statistical heterogeneity. If comparing groups, describe the direction of the effect. | The article has been supplemented |
|  | 20c | Present results of all investigations of possible causes of heterogeneity among study results. | Page 9, line 248-254; pages 14-15, line 391-402 |
|  | 20d | Present results of all sensitivity analyses conducted to assess the robustness of the synthesized results. | The article has been supplemented |
| Reporting biases | 21 | Present assessments of risk of bias due to missing results (arising from reporting biases) for each synthesis assessed. | Page 12, line 331-335 |
| Certainty of evidence | 22 | Present assessments of certainty (or confidence) in the body of evidence for each outcome assessed. | Page 12, line 331-335 |
| **DISCUSSION** | | |  |
| Discussion | 23a | Provide a general interpretation of the results in the context of other evidence. | Pages 12-13, line 338-343 |
|  | 23b | Discuss any limitations of the evidence included in the review. | Page 15, line 408-417 |
|  | 23c | Discuss any limitations of the review processes used. | Page 15, line 408-417 |
|  | 23d | Discuss implications of the results for practice, policy, and future research. | Page 15, line 418-422 |
| **OTHER INFORMATION** | | |  |
| Registration and protocol | 24a | Provide registration information for the review, including register name and registration number, or state that the review was not registered. | Page 5, line 115 |
|  | 24b | Indicate where the review protocol can be accessed, or state that a protocol was not prepared. | Page 5, line 115 |
|  | 24c | Describe and explain any amendments to information provided at registration or in the protocol. | Page 5, line 115 |
| Support | 25 | Describe sources of financial or non-financial support for the review, and the role of the funders or sponsors in the review. | Page 16, line 434-439 |
| Competing interests | 26 | Declare any competing interests of review authors. | Page 16, line 442 |
| Availability of data, code and other materials | 27 | Report which of the following are publicly available and where they can be found: template data collection forms; data extracted from included studies; data used for all analyses; analytic code; any other materials used in the review. | Page 16, line 449-452 |

*From:*  Page MJ, McKenzie JE, Bossuyt PM, Boutron I, Hoffmann TC, Mulrow CD, et al. The PRISMA 2020 statement: an updated guideline for reporting systematic reviews. BMJ 2021;372:n71. doi: 10.1136/bmj.n71

For more information, visit: <http://www.prisma-statement.org/>
